# Unraveling How Membrane Nanostructure Changes Impact the Eye Irritation of Nonionic Alkyl Ethoxylate Surfactants

**DOI:** 10.1021/acsami.3c14794

**Published:** 2023-12-11

**Authors:** Xuzhi Hu, Mingrui Liao, Kangcheng Shen, Ke Ding, Mario Campana, Sophie van der Kamp, Elizabeth F. McInnes, Faheem Padia, Jian R. Lu

**Affiliations:** † Biological Physics Group, Department of Physics and Astronomy, School of Natural Sciences, University of Manchester, Oxford Road, Manchester M13 9PL, U.K.; ‡ Rutherford Appleton Laboratory, STFC ISIS Facility, Didcot OX11 0QX, U.K.; § Jealott’s Hill International Research Centre, Syngenta, Bracknell, Berkshire RG42 6EY, U.K.

**Keywords:** lipid bilayer, membrane nanostructures, cytotoxicity, agri-sprays, neutron reflection and scattering, membrane disruption

## Abstract

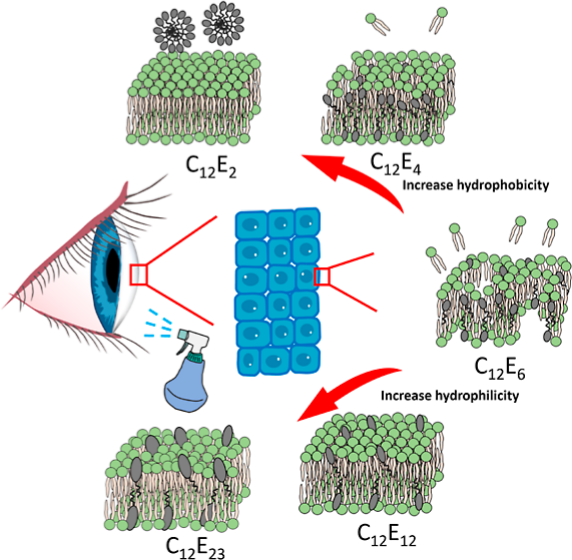

Nonionic surfactants used in agri-spraying processes may cause varying degrees of corneal irritation when they come in direct contact with farmers’ eyes, and the exact irritations are thought to be determined by how surfactants interact with corneal cell membranes. However, how nonionic surfactants interact with cell membranes at the molecular and nano levels remains largely unexplored. In this study, the interactions between nonionic surfactants (alkyl ethoxylate, C_12_E_*m*_) and lipid membranes were examined by membrane permeability measurement, quartz crystal microbalance with dissipation, dual polarization interferometry, confocal laser scanning microscopy, and neutron reflection, aiming to reveal complementary structural features at the molecular and nano levels. Apart from the extremely hydrophobic surfactant C_12_E_2_, all nonionic surfactants studied could penetrate the model cell membrane composed of a phosphocholine lipid bilayer. Nonionic surfactants with intermediate amphiphilicity (C_12_E_6_) rapidly fused into the lipid membrane and stimulated the formation of pores across the lipid bilayer, consistent with the cytoplasm leakage and fast cell necrosis observed from the cytotoxicity study of corneal cells. In comparison, while hydrophobic and hydrophilic surfactants [those with long and short ethoxylates (C_12_E_4,12,23_)] could cause mild structural alteration to the outer lipid layer of the membrane, these structural changes were insufficient to elicit large cytoplasmic leakage rapidly and instead cell death occurred over longer periods of time due to changes in the membrane permeability. These results reveal the strong link of surfactant–lipid membrane interactions to surfactant cytotoxicity and the association with amphiphilicity of nonionic surfactants.

## 1 Introduction

Commercial pesticide products are often formulated as emulsifiable concentrates in which nonionic surfactants are widely used for various purposes.^1−4^ Nonionic surfactants are generally composed of a hydrophobic tail and a hydrophilic ethoxylate group. When used in agri-spraying processes, first, they help to redisperse the concentrated formulation upon dilution in the farmers’ spray tank.^5^ Next, they can help to reduce bouncing of spray droplets as they interact with the leaf surface.^6−9^ Finally, nonionic surfactants can also serve as adjuvants to improve droplet coverage on leaves,^9,10^ pesticide solubility and mobility^11−13^ in the deposit, and consequently uptake into the plant.^14−17^ However, it has been shown that irritations can occur when nonionic surfactants are accidentally placed in contact with end-users’ eyes during spraying.^18^ Thus, there is an ever-pressing need to understand how nonionic surfactants may irritate corneal cells. This knowledge can help the agrochemical industry design safer, less irritating nonionic surfactants by minimizing the cytotoxicity while maximizing their benefits for leaf dispersal.

Exposure of cells to surfactants can cause cell death through necrosis.^19^ Although the exact structural and morphological processes underlying surfactant-induced necrosis remain largely unexplored, several studies have reported the loss of plasma membrane integrity and leakage of cellular contents into the extracellular space, causing inflammatory responses.^20^ Our previous studies have demonstrated that the cytotoxicity of nonionic ethoxylated surfactants is determined by their critical micellar concentrations (CMCs).^21^ Most surfactants cause cytotoxicity to cells by disrupting cell membranes and subsequently causing the leakage of the cytoplasm, showing that the cytotoxic responses are time- and concentration-dependent.^22^ Morphological changes of cytoplasmic membranes often proceed disruptions of other cell organelles associated with swelling of the endoplasmic reticulum (ER) and mitochondria and functional delay of the Golgi apparatus,^23,24^ but how nonionic surfactants affect their cytotoxicity toward human epithelium cells and the roles of their membrane-lytic interactions are less studied. Moreover, the impact of the surfactants on changes in the membrane nanostructure and composition requires further characterization. This is the focus of this study.

Hydrophobically balanced surfactants are expected to impose cytotoxicity through a fast cell membrane disruption. Extremely hydrophobic surfactants (those with long hydrocarbon tail chains or short ethoxylate head lengths) and hydrophilic surfactants (those with long ethoxylate head lengths) may have relatively low cytotoxicity associated with their different modes of action with the membranes. Previous studies have revealed that hydrophobic surfactants do not interact strongly with cell lipid membranes while hydrophilic surfactants induce increased cell membrane permeability and compromise the cytoplasm.^22^ As surfactant cytotoxicity strongly relates to the interaction between the surfactant and the cell membrane, it is of great importance to gain a better understanding of the morphological transitions and the mechanistic processes. In particular, the structure and composition of the cell membrane before, during, and after exposure to different nonionic surfactants would provide useful insights into the cytotoxicity mechanism.

In this study, the nanostructures of the membranes upon exposure to a group of nonionic surfactants (alkyl ethoxylates, denoted as C_*n*_E_*m*_), are unraveled using both natural and model cell membranes. A homologous set of C_*n*_E_*m*_ surfactants with a fixed tail length of C12-(dodecyl) but varied ethoxylate units (C_12_E_2,4,6,12, and 23_) was studied. Cell membrane permeability measurements were combined with several physicochemical characterizations from lipid membrane models with and without nonionic surfactants, including the direct observation of nonionic surfactants across the natural mammalian cell membranes. Supported lipid bilayer (SLB) models were used to facilitate measurement of the insertion of nonionic surfactants into lipid membranes by quartz crystal microbalance with dissipation (QCM-D), dual polarization interferometry (DPI), confocal laser scanning microscopy (CLSM), and neutron reflection (NR). The detailed structural configurations provided a useful basis to link the membrane-lytic processes to the cytotoxicity of different nonionic surfactants, which is important to understand both the design and selection of surfactants for agrochemical products.

## 2 Results and Discussion

### 2.1 Morphological and Cell Viability Changes Due to the Binding of C_12_E_*m*_

**Figure 1:**
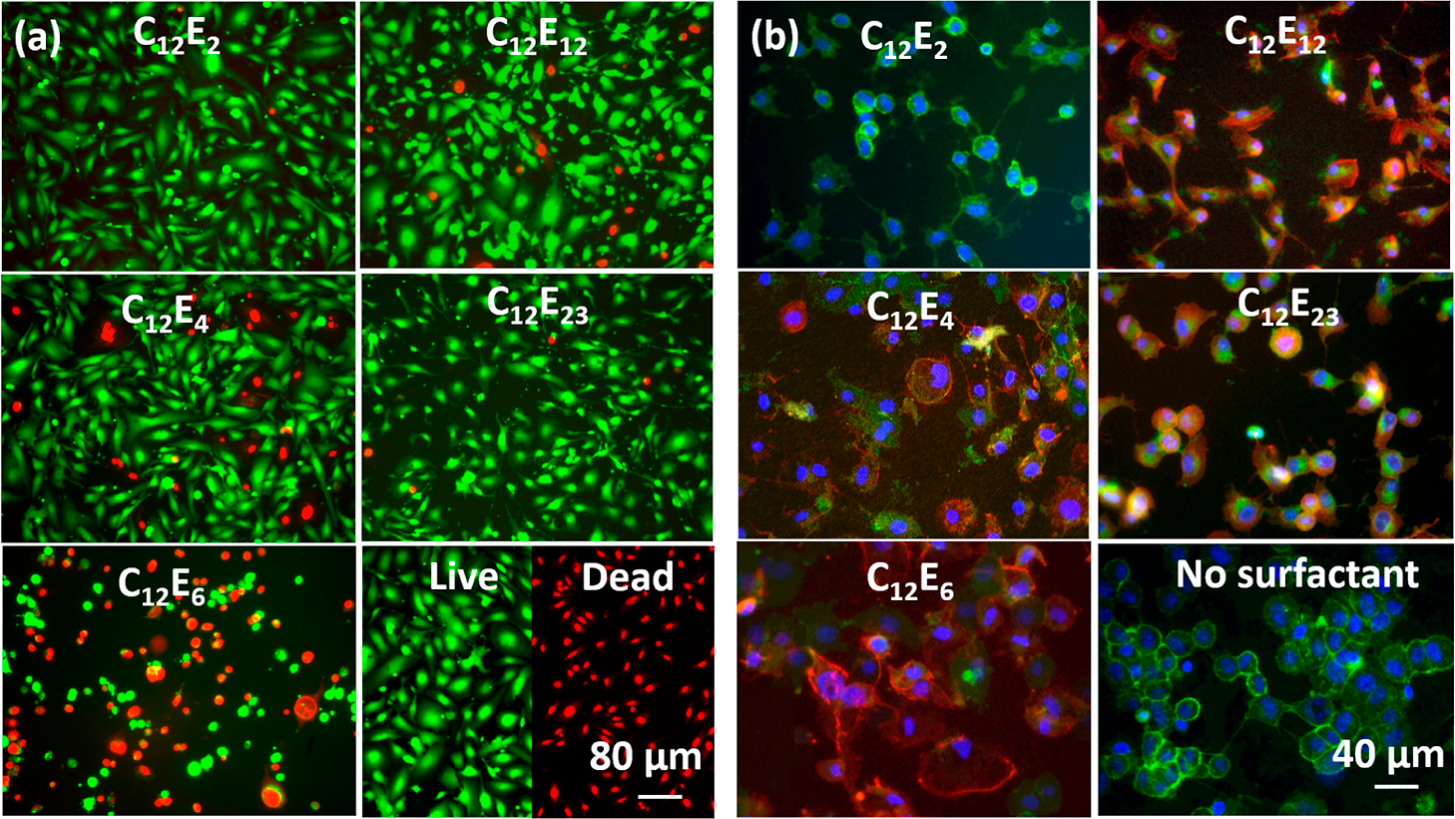
(a) HCE cell viability and morphological changes against C_12_E_2_, C_12_E_4_, C_12_E_6_, C_12_E_12_, and C_12_E_23_ for 30 min at a concentration of 1 CMC. Live cells were stained in green. Dead cells were stained in red. (b) Morphologies for HCE cells exposed to C_12_E_2_, C_12_E_4_, C_12_E_6_, C_12_E_12_, C_12_E_23_, and no surfactant for 30 min. Cell nuclei (blue), cytoplasm (green), and cytoskeleton (red) were stained by Hoechst, plasma membrane indicator 488, and Phalloidin 594, respectively. As Phalloidin 594 is not a cell membrane-permeable stain, the cytoskeleton is stained due to either the cell membrane being permeable or disrupted by the surfactant.

The membrane morphological and cell viability changes of human corneal epithelial (HCE) cells against varied nonionic surfactants are studied by the live and dead assay, with representative images of HCE cells in response to different surfactants after 30 min exposure at a concentration of 1 CMC being illustrated in Figure 1a (cell concentration: 2 × 10^5^/mL). The further use of fluorescent dyes allowed the different membrane lysis processes of the surfactants to be elucidated, as illustrated in Figure 1b. To gain better fluorescent signals, the cell concentration used was 5 × 10^4^/mL. Images from individual color channels were recorded and then merged. Hoechst and plasma stain 488 can penetrate the cell membrane, while Phalloidin 594 is not a cell membrane-permeable stain. Thus, the stained cytoskeleton indicates that the cell membrane is either disrupted or permeable.

After 30 min of exposure, C_12_E_6_ clearly caused rapid leakage of most cytoplasmic fluids as the green fluorescence was weak while the cytoskeleton was stained, indicating that C_12_E_6_ disrupted the cell membrane. As a result, C_12_E_6_ killed more than half of the total cells. C_12_E_4_, C_12_E_12_, and C_12_E_23_ enhanced the cell membrane permeability as the cytoskeleton was stained; however, the structural changes of the cell membranes were not sufficient to elicit large cytoplasmic leakage as the signal for the cytoplasm was still relatively strong. Thus, most cells are still alive. In addition, a very weak red fluorescent signal was detected for C_12_E_2_, indicating that the cell membrane permeability was not changed by C_12_E_2_, leading to almost no cell death. These observations are consistent with our previous cytotoxicity studies of nonionic surfactants,^25^ indicating that nonionic surfactant cytotoxicity is determined by their interaction with the cell membrane, which is dominated by the different ethoxylate head sizes.

### 2.2 Mechanistic Kinetics of C_12_E_*m*_ Micelles Binding to the Model Lipid Membrane

**Figure 2:**
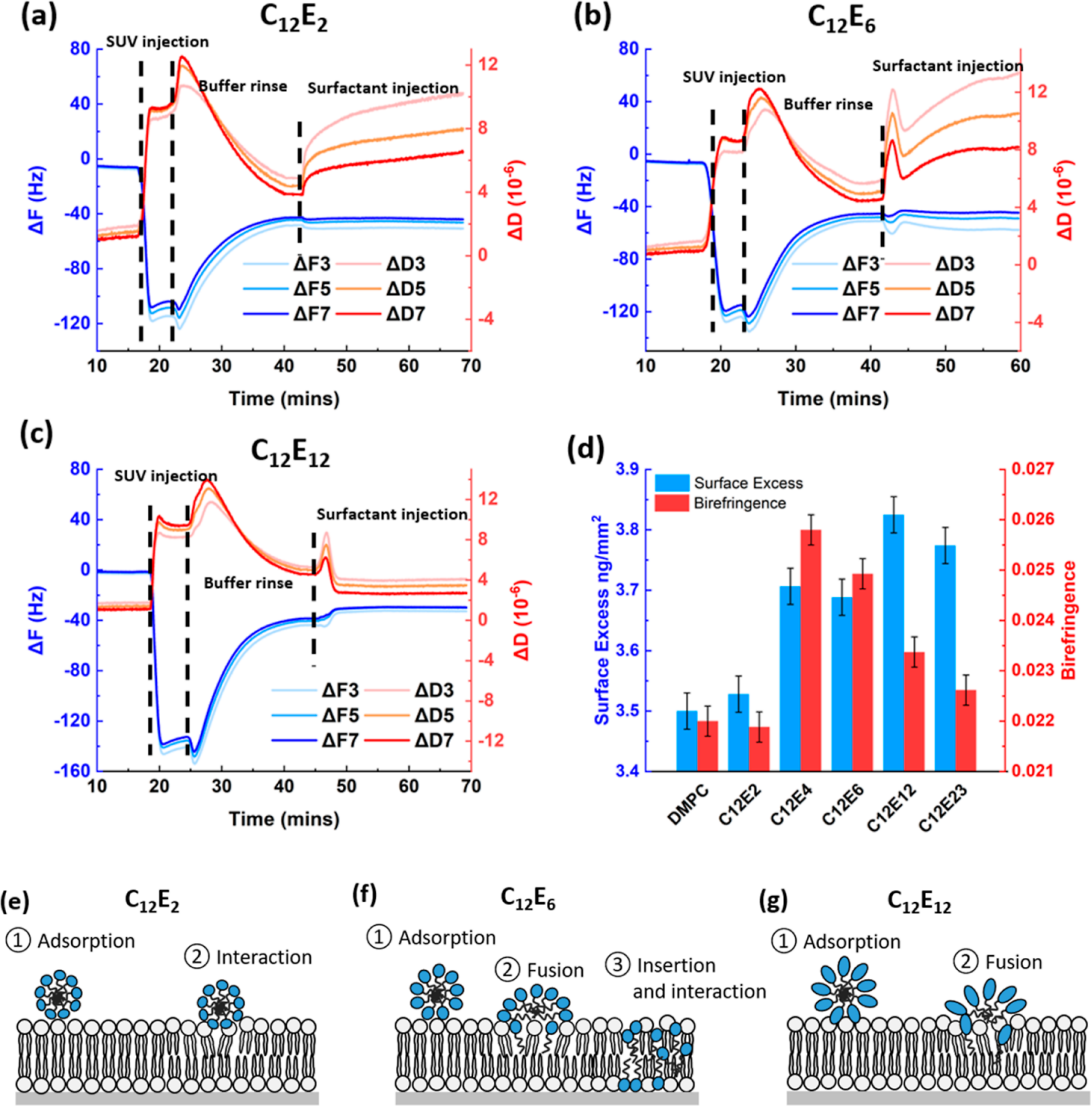
Resonance frequency overtones Δf and the energy dissipation overtones Δ*D* (*n* = 3^rd^, 5^th^, and 7^th^) over time for (a) C_12_E_2_, (b) C_12_E_6_, and (c) C_12_E_12_ at a concentration of 1 CMC. (d) Surface excess change and birefringence of the model DMPC bilayer during surfactant binding. Cartoon showing the kinetics of (e) C_12_E_2_, (f) C_12_E_6_, and (g) C_12_E_12_ binding with DMPC bilayers.

The binding kinetics of C_12_E_*m*_ micelles to the model 1,2-dimyristoyl-*sn*-glycero-3-phosphocholine (DMPC) SLB was traced dynamically using QCM-D. By examination of the change of the DMPC SLB vibration frequency and energy dissipation, the properties of the adsorbed layer in terms of the total weight and rigidity can be determined. Figure 2a–c shows the shift of the sensor’s resonance frequency harmonic overtones Δ*f* and the energy dissipation harmonic overtones Δ*D* (*n* = third, fifth, and seventh) over time for C_12_E_2_, C_12_E_6_, and C_12_E_12_ at a concentration of 1 CMC, respectively. Figure S1a,b shows the profiles of Δ*f* and Δ*D* for C_12_E_4_ and C_12_E_23_ at the same concentration, respectively. The bare DMPC bilayer was first formed via the fusion method. The rapid increase in energy dissipation and drop in frequency indicated the adsorption of DMPC small unilamellar vesicles (SUVs) onto the chip. The SUVs were fused to form a bilayer with the buffer rinse and the unfused SUVs were removed. The bare DMPC bilayer has a Δ*f* of roughly −40 Hz and an Δ*D* of about 3–5 × 10^–6^.

After rinsing with plenty of buffer, when the frequency and energy dissipation signals reached equilibrium, the C_12_E_*n*_ surfactants were injected into the DMPC SLB. The kinetic mechanisms for different surfactants interacting with the model lipid bilayer varied significantly. Slight changes in the frequency were observed for all C_12_E_*n*_ surfactants, indicating that the total weight of the adsorbed layer did not change dramatically. Apart from the C_12_E_2_ interaction, fluctuations in energy dissipation were observed for C_12_E_4_, C_12_E_6_, C_12_E_12_, and C_12_E_23_ surfactants, indicating that these micelles rapidly fused into the DMPC lipid bilayers upon adsorption. Further great separation in the overtones of the energy dissipation was observed for C_12_E_4_ and C_12_E_6_ injection, indicating that the rigidity of the DMPC bilayer was changed due to the surfactant insertion.

The surface excess change and birefringence of the model DMPC bilayer during surfactant binding were traced by DPI. The surface excess of the bare DMPC lipid bilayer was found to be 3.5 ng/mm^2^. The birefringence of the bare DMPC lipid bilayer was 0.022. These values are broadly consistent with previous measurements,^26^ indicating that the bare DMPC SLB is a smooth and uniform layer and the DMPC molecules are well-ordered in the SLB. The lipid bilayers were then exposed to C_12_E_*m*_ at the concentration of 1 CMC for 3 min.

As shown by Figure 2d, with the exception of C_12_E_2_, all surfactants bind or adsorb onto the lipid bilayer as the weights all increase. The total surface excess increased to 3.7 ng/mm^2^ for C_12_E_4_ and C_12_E_6_ and to 3.80 ng/mm^2^ for C_12_E_12_ and C_12_E_23_. Although the changes in the surface excesses are greater for C_12_E_12_ and C_12_E_23_, the associated changes in birefringence are much smaller for these surfactants. The birefringence increases by only 0.001 units for C_12_E_12_ and C_12_E_23_ while the birefringence increases by 0.004 units for C_12_E_4_ and 0.003 units for C_12_E_6_, respectively. In other words, despite the lower amounts of C_12_E_4_ and C_12_E_6_ being inserted into the DMPC bilayer, these surfactants have a significant effect on birefringence. This implies that these surfactants are increasing the ordering of the DMPC bilayer. C_12_E_12_ and C_12_E_23_, on the other hand, are likely to mainly adsorb onto the outer surface of the lipid bilayer and only elicit slight alterations to the bilayer structure.

Thus, the hydrophobicity of the nonionic surfactants plays an important role in determining their interacting kinetics with the lipid bilayer. Short EO (C_12_E_2_) can only weakly adsorb onto the lipid bilayer and have little effect on the lipid structures (Figure 2e). Amphiphilic C_12_E_4_ and C_12_E_6_ micelles quickly fuse into the lipid bilayer, with further insertion after the fusion process (Figures 2f and S1c). Long EO (C_12_E_12_ and C_12_E_23_) can also fuse into the lipid bilayer but the surfactant molecules are trapped in the outer surface of the lipid bilayer (Figures 2g and S1d).

### 2.3 Morphologies, Phase Behaviors, and Structures of the Lipid Membrane with/without Nonionic Surfactants

**Figure 3:**
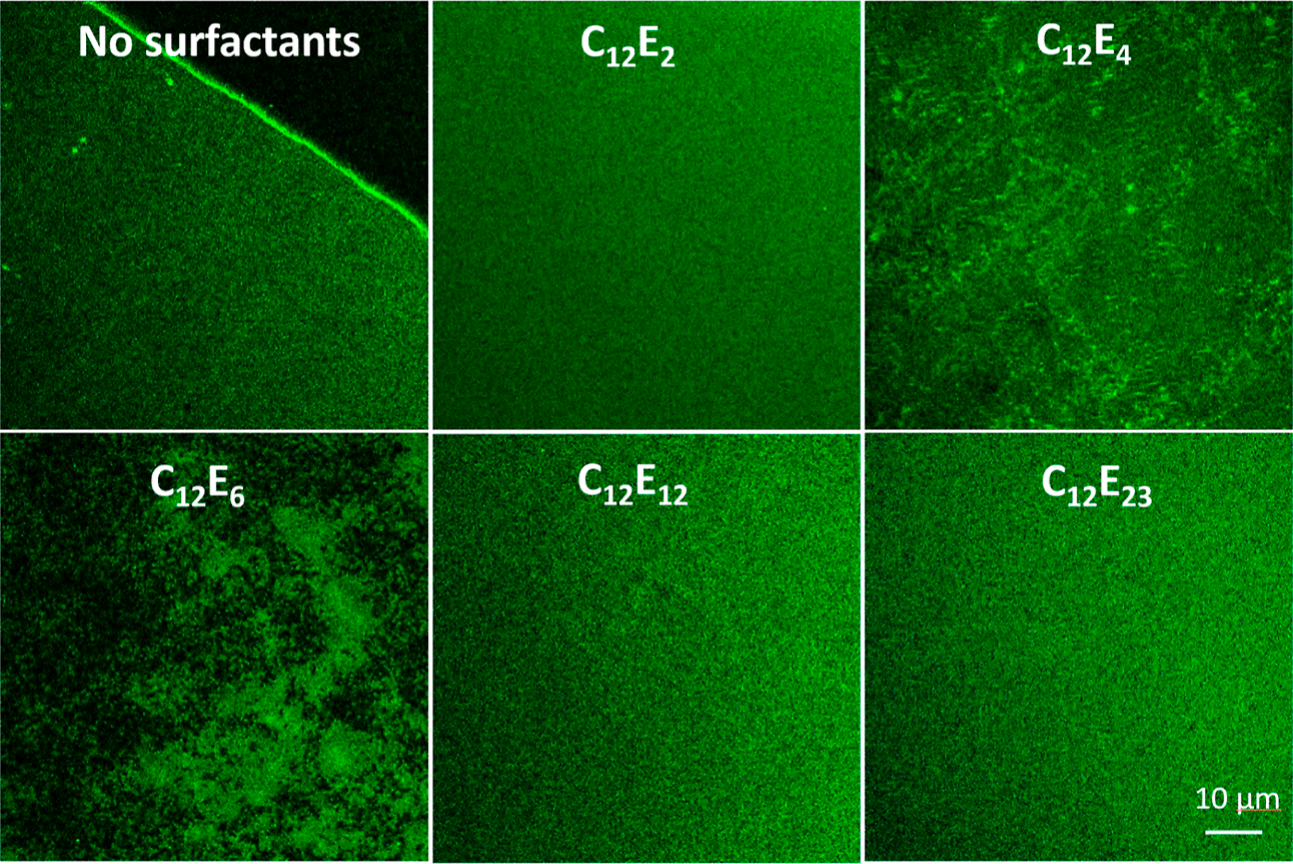
Representative CLSM images of the C12-HPC SLB in the absence and presence of C_12_E_2_, C_12_E_4_, C_12_E_6_, C_12_E_12_, and C_12_E_23_ (1 CMC). Insignificant interaction was observed for short EO and long EO surfactants (C_12_E_2_, C_12_E_12_, and C_12_E_23_). C_12_E_4_ restructured the membrane, increasing the membrane inhomogeneity. C_12_E_6_ largely removed the lipids, forming pores within the membrane, which are responsible for rapid cytoplasm leakage and cell necrosis.

The morphologies of a fluorescent lipid membrane in the absence and presence of different nonionic surfactants (1 CMC) were followed by CLSM, with representative images being shown in Figure 3. The C12-HPC lipids clearly formed a membrane structure on the glass surface. Although the exact thickness of the membrane cannot be accurately obtained, the main morphological features of the exposed C12-HPC membrane are consistent with previous observations,^27^ indicating the high lateral homogeneity of the lipid membrane.

Neither short EO nor long EO surfactants (C_12_E_2_ or C_12_E_12_ and C_12_E_23_) caused observable changes to the lipid membrane, indicating minimal disruptions to the lipid membrane. In contrast, C_12_E_4_ clearly restructured the membrane, leading to an increased inhomogeneity in the distribution of the fluorescent lipids within the membrane. The structural alteration is likely to occur to the outer lipid leaflet as no observable pores are formed due to the restructuring. However, a large amount of lipid is removed by C_12_E_6_. A large amount of lipid removal visibly leads to the formation of transmembrane pores within the membrane across all scanned areas, which are responsible for cytoplasm leakage and cell necrosis.

**Figure 4:**
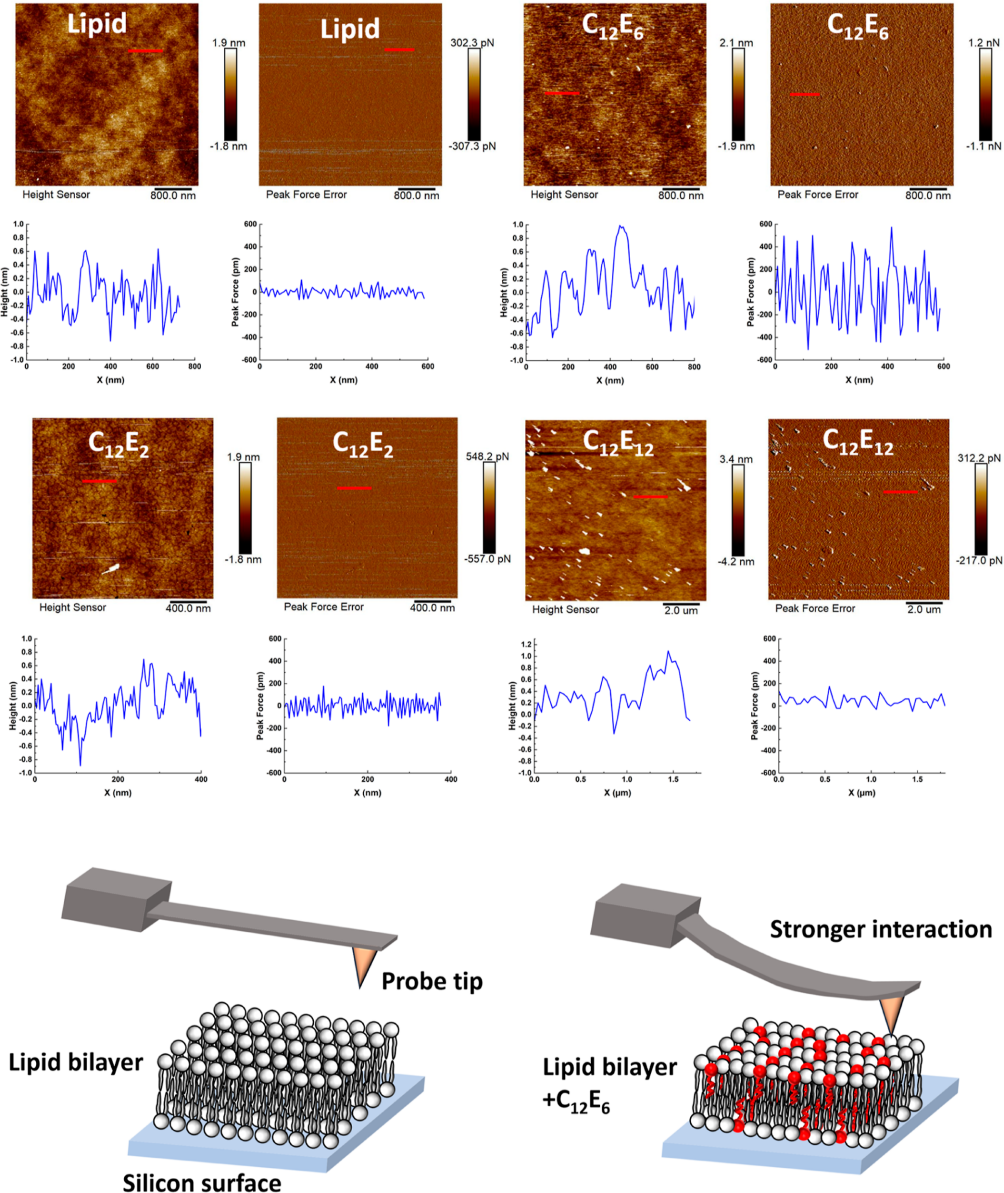
AFM images of lipid membranes in the absence and presence of C_12_E_2_, C_12_E_6_, and C_12_E_12_ surfactants in terms of height and peak force error. Schematics show that C_12_E_6_ surfactant insertion made lipid molecules more packed, leading to stronger interactions between the lipid membrane and the AFM probe tip.

The phase behaviors and structures of the DMPC lipid membrane formed onto the silicon wafer surface in the absence and presence of different nonionic surfactants (1 CMC) were characterized by liquid AFM at the liquid/solid interface, with representative images being shown in Figures 4 and S2. The lipid bilayer formed was very uniform with the height fluctuation being within ±0.5 nm and the peak force error being within ±100 pm across all scanned areas.

Upon C_12_E_6_ binding, some clusters were formed on the lipid membrane as shown by the white spots in Figure 4, indicating the removal of lipids by surfactants. No obvious changes in the height fluctuations were observed. However, the peak force error increased significantly to ±600 pm, indicating that the number of molecules per unit area increased greatly due to surfactant insertion.^28^ Hydrophobic surfactants C_12_E_2_ caused no changes to the lipid membrane morphology and phase. Due to the strong interaction between the long EO surfactant and the AFM tip, the scanning area can only be obtained at a larger scale. Hydrophilic surfactants C_12_E_12_ slightly increased the height of the lipid membrane, but the peak force error remained unchanged, indicating the insertion of a small amount of surfactants into the lipid membrane. As shown in Figure S2, C_12_E_4_ caused similar changes to the lipid membrane compared to C_12_E_6_, while C_12_E_23_ increased the height of the lipid membrane but caused insignificant changes to the peak force error.

Together with the measurements of changes in the total weight of the adsorbed materials from QCM-D and DPI, the CLSM and AFM images of the SLB in the absence and presence of different surfactants demonstrated the varied interaction patterns at the *xy*-plane. However, it is still difficult to gain the detailed structural changes of the SLB upon surfactant binding in terms of composition and structural configuration.

### 2.4 Nanostructural Changes of the SLB in the Absence and Presence of Nonionic Surfactants

**Figure 5:**
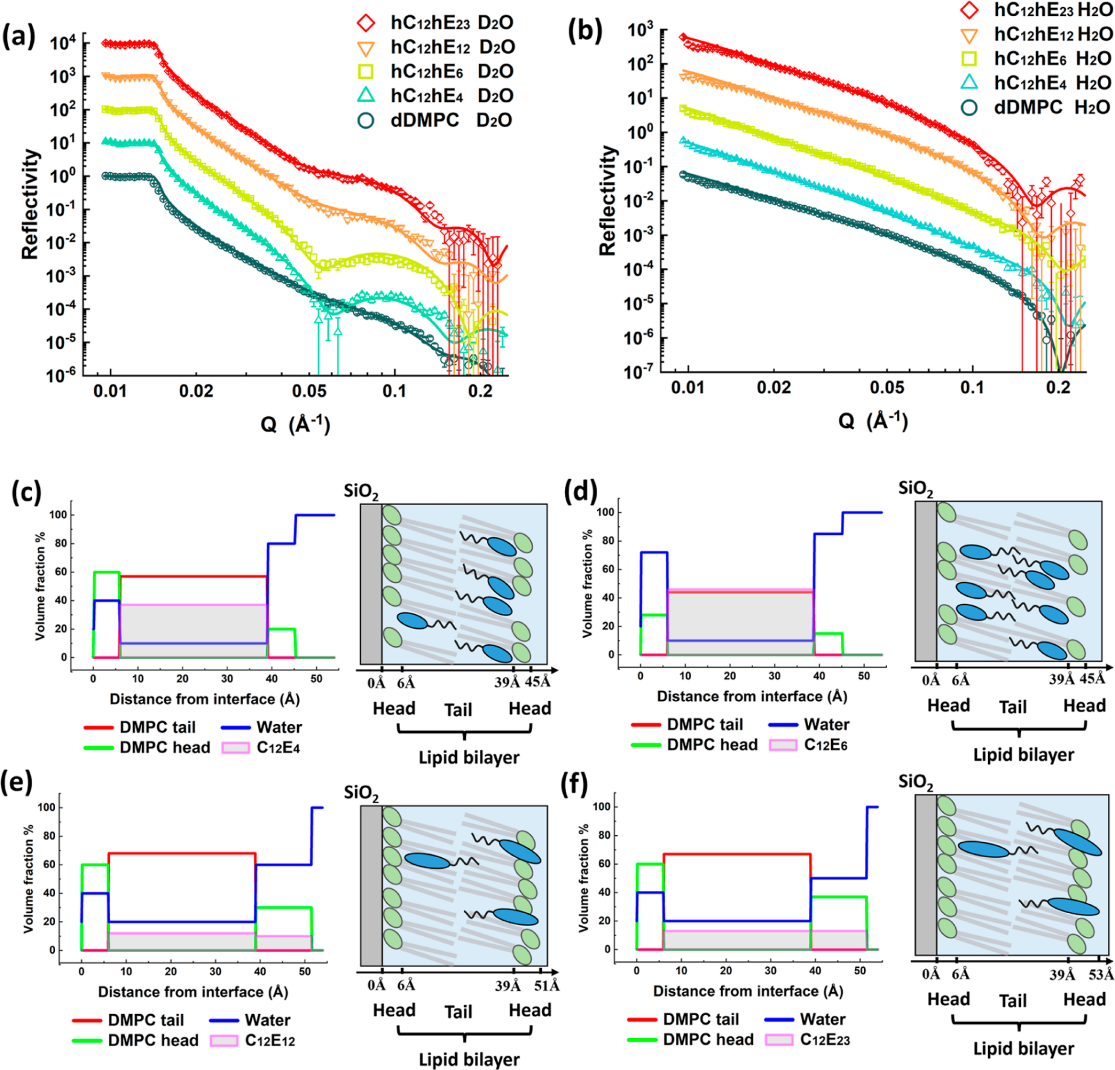
NR profiles for (a) dDMPC with hC_12_hE_4,6,12,23_ in D_2_O and (b) dDMPC with hC_12_hE_4,6,12,23_ in H_2_O. For better visualization, the profiles are shifted vertically by 1, 10, 100, 1000, and 10,000 from bottom to top. Volume fraction profiles and schematics for (c) C_12_E_4_, (d) C_12_E_6_, (e) C_12_E_12_, and (f) C_12_E_23_.

The detailed structures of the DMPC SLB in the absence and presence of nonionic surfactants were studied by NR. As there is nearly no interaction between C_12_E_2_ and DMPC SLB, neutron experiments were performed on C_12_E_4–23_. The NR profiles for D_2_O and H_2_O contrasts are presented in Figure 5a,b, respectively. Notably, for D_2_O, the scattering length density (SLD) for the deuterated DMPC tail (6.9 × 10^–6^ Å^–2^) is very close to D_2_O (6.35 × 10^–6^ Å^–2^) and only surfactant (0 × 10^–6^ Å^–2^) aggregation across the SLB can stimulate NR profile change. Similarly, H_2_O and hydrogenated surfactants are indistinguishable to NR and only the distribution of the deuterated DMPC tail in response to surfactant binding can alter the NR profile. All of the detailed fitting parameters are listed in Table 1.

**Table 1:** Detailed Best-Fit Parameters for NR Profiles for the DMPC SLB on Silicon Oxide Wafers in the Absence and Presence of C_12_E_*m*_ in D_2_O and H_2_O^a^

			Si	SiO_2_	layer 1 (inner head)	layer 2 (tail)	layer 3 (outer head)	solvent
dDMPC	thickness		∞	14	6	33	6	∞
	SLD	D_2_O	2.07	3.9	3.0	6.8	3.0	6.35
		H_2_O		2.7	0.2	5.4	0.2	–0.56
+hC_12_hE_4_	thickness		∞	14	6	33	6	∞
	SLD	D_2_O	2.07	3.9	3.0	4.0	5.0	6.35
		H_2_O		2.7	0.2	3.6	–0.4	–0.56
+hC_12_hE_6_	thickness		∞	14	6	33	6	∞
	SLD	D_2_O	2.07	3.9	4.78	3.65	5.51	6.35
		H_2_O		2.7	–0.2	3.65	–0.36	–0.56
+hC_12_hE_12_	thickness		∞	14	6	33	12	∞
	SLD	D_2_O	2.07	3.9	3.0	5.9	4.1	6.35
		H_2_O		2.7	0.2	4.5	1.7	–0.56
+hC_12_hE_23_	thickness		∞	14	6	33	14	∞
	SLD	D_2_O	2.07	3.9	3.0	6.0	3.8	6.35
		H_2_O		2.7	0.2	4.2	2.3	–0.56

a The thickness for each layer is in the unit of ±1 Å. The SLD is in the unit of ×10^–6^ Å^–2^ (±3%). ∞ represents infinity.

The silicon oxide surface was first characterized in D_2_O. The silicon oxide is a uniform layer comprising 80 vol % SiO_2_ and 20 vol % hydration with a thickness of 14 Å. The DMPC SLB was formed onto the silicon oxide surface at 40 °C and characterized in D_2_O and H_2_O. To avoid temperature effects on the physiochemical properties of nonionic surfactants, the temperature was then decreased to 20 °C before any surfactant injection and the SLB was characterized in D_2_O and H_2_O at 20 °C again.

The exposed DMPC SLB was fitted into a “sandwich-like” 3-layer model, representing an inner lipid head, a middle tail region, and outer head groups, respectively. The fitted results for the exposed DMPC lipid bilayer are shown in Figure S3 (a: the SLD profile against distance; b: the converted volume fraction for DMPC tail, head, and water against distance). The DMPC lipid molecules form a well-packed dense bilayer. Both inner and outer head layers are 6 Å in thickness and contain 40 vol % water. The tail layer is 33 Å in thickness and contains 20 vol % water. As the extended length of the DMPC tail is 19–20 Å and the extended length for a PC head is roughly 10 Å,^29,30^ the DMPC heads are inclined in an angle of 37° against the surface. Assuming there is no overlap of DMPC tails in the SLB, the angle between the DMPC tail and surface is 55°, indicating that there is an angle of 18° between the DMPC head and tail. The density of DMPC is estimated as 1.04 g/cm^3^ (molar weight: 678 g/mol, molar volume: 1105 Å^3^). Thus, the adsorbed weight for the bare DMPC SLB can be easily calculated as 3.53 ng/mm^2^, consistent with our DPI results.

Due to the adsorption and penetration of nonionic surfactant molecules, the NR profiles have been altered. The best-fit results of the SLD profiles against distance from the interface and the calculated volume fraction profiles for the lipid tail, lipid head, water, and surfactants for different surfactants are shown in Figures S4 and 5c–f.

The C_12_E_4_ molecules got well-inserted into the DMPC tail layer, with the C_12_E_4_ volume fraction being roughly 40 vol %. The tail volume fraction decreases from 80 vol % to less than 60 vol %, indicating that some lipids are removed. The removed lipids are mainly from the outer leaflet, as the inner lipid head structure remains unchanged while the lipid head volume fraction in the outer leaflet decreases by roughly 40%. As the thickness for different layers of the SLB remained the same, the orientations of lipid molecules were not changed upon C_12_E_4_ insertion.

The C_12_E_6_ molecules got inserted into the DMPC bilayer, as well, and the SLB structure was changed greatly. The surfactants occupy roughly 50 vol % in the DMPC tail layer. Both inner and outer lipid molecules are removed as the volume fraction of the lipid head in the inner layer decreases by 30 vol % and the outer layer decreases by a slightly greater value (approximately 40 vol %), indicating that the integrity of the lipid membrane was significantly affected and nanopores must be formed in the SLB. The thickness for each layer did not change, indicating that the orientations of the lipid molecules were not changed by the interaction with C_12_E_6_.

Longer EO surfactants like C_12_E_12_ and C_12_E_23_ also got inserted into the lipid bilayer but only occupied approximately 10 vol % and the lipid tail volume fraction decreased slightly, indicating that the main structures of the lipid bilayer were not significantly changed. The thickness of the outer head layer expands to 12 and 14 Å for C_12_E_12_ and C_12_E_23_, respectively, indicating that the orientation of the outer lipid was likely changed and some C_12_E_12_ and C_12_E_23_ molecules were adsorbed onto the bilayer.

### 2.5 Link between Lipid–C_*n*_E_*m*_ Interaction and Nonionic Surfactant Eye Irritancy

Nonionic surfactants such as C_12_E_6_ at or above CMC with intermediate amphiphilicity kill cells very rapidly.^25^ The cytotoxicity (at the same concentrations) is reduced in terms of a longer killing time and higher cell viability when surfactant hydrophobicity is either increased or reduced significantly. C_12_E_4_, C_12_E_12_, and C_12_E_23_ exhibited observable cytotoxicity after 24 h. The different cytotoxicities for surfactants with varied hydrophobicity arise from their distinctly different membrane-lytic ability. Nonionic surfactants with intermediate amphiphilicity bear strong membrane-lytic power and elicit large cytoplasm leakage. In contrast, hydrophobic surfactants adsorb onto cell membranes with minimal structural damage. Hydrophilic surfactants can also adsorb onto cell membranes but cause mild structural changes, and these changes are insufficient to elicit large cytoplasmic leakage over a short period of time. Clearly, the way that nonionic surfactants interact with the lipid membrane determines their necrosis ability and cytotoxicity toward human corneal cells and thus their potential to induce eye irritation.

In this work, the detailed structural configurations of the lipid membrane upon binding of nonionic surfactants with different hydrophobicities are unraveled at the molecular level, with clear evidence indicating the insertion of different nonionic surfactants into the lipid membrane. The intermediate amphiphilic C_12_E_6_ is strongly inserted into the lipid membrane and causes the strongest removal of the lipids from the bilayer. As a result, transmembrane pores are formed across the lipid membrane, consistent with the large and rapid cytoplasm leakage. While C_12_E_4_ can also get heavily inserted into the lipid membrane and cause strong lipid removal, the structural changes mainly occur at the outer leaflet of the lipid membrane, indicating that C_12_E_4_ molecules are unable to break the inner hydrophilic lipid head boundary. The inner leaflet structure remains unchanged so that no large cytoplasm leakage happens. Nevertheless, the membrane permeability is altered. More hydrophobic surfactants such as C_16_E_6_ can only adsorb onto the cell membrane in the static situation but no interaction happens.^25^ The hydrophobic surfactant C_12_E_2_ hardly adsorbs onto the lipid bilayer, especially under dynamic flow, indicating that the binding of hydrophobic surfactant micelles with the lipid membrane is very weak.

Our findings on the nanostructural changes of the cell membrane against varied nonionic surfactants correlate with eye irritation studies. Our results are consistent with previous reports that the hemolytic activity of nonionic surfactants decreases with the increased carbon numbers in the hydrophobic tail and increased EO numbers.^31^ These results are also broadly consistent with the commercial rabbit eye test reports published by the European Committee of Organic Surfactants and their Intermediates (CESIO), which suggest for a given surfactant tail length, shorter EO (≤4 EO) and longer EO (>15 EO) lengths cause mild eye irritation while the intermediate EO units cause severe eye damage.^32^

**Figure 6:**
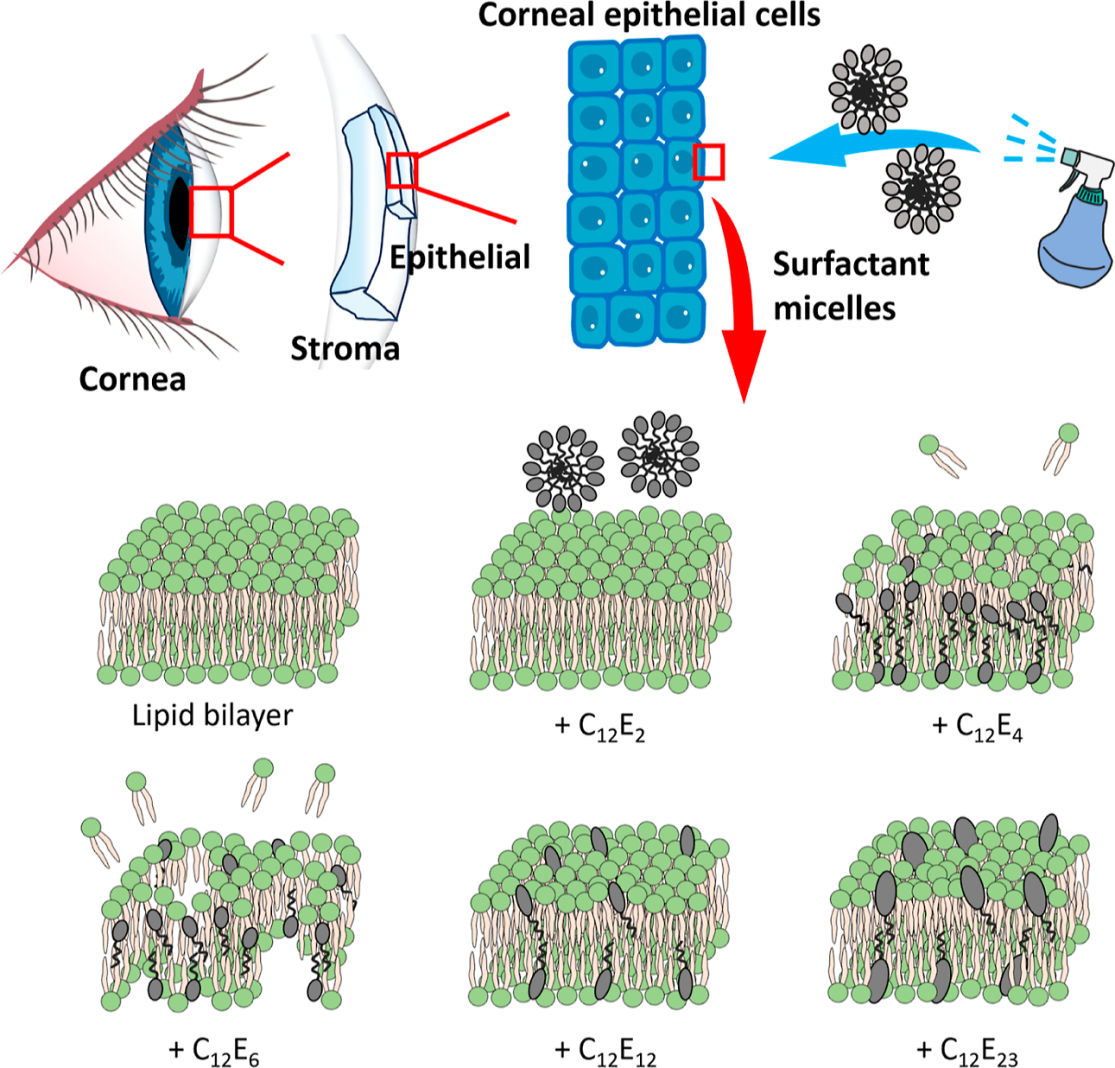
Schematic cartoon showing the different surfactant–lipid bilayer interactions for C_12_E_2_, C_12_E_4_, C_12_E_6_, C_12_E_12_, and C_12_E_23_. No interaction is observed for C_12_E_2_. C_12_E_4_ gets largely inserted into the lipid bilayer, but the structural changes mainly occur at the outer leaflet, causing mild disruption on membrane integrity but not sufficient to elicit cytoplasm leakage in a short period. C_12_E_6_ largely removed lipids from the lipid bilayer, and nanopores were formed, leading to cytoplasm leakage. Hydrophilic surfactants C_12_E_12_ and C_12_E_23_ can be inserted into the lipid bilayer, but the amount that gets inserted is small. They also adsorb onto the lipid bilayer, causing some mild structural change on the top lipid leaflet.

The structural changes of the lipid membrane in the absence and presence of nonionic surfactants with varied hydrophobicity can be schematically outlined in Figure 6. Surfactants with well-balanced hydrophobicity tend to accumulate in the lipid bilayer, especially in the hydrophobic core region, eliciting major toxicity and cell death. However, excessively hydrophobic surfactants cannot break the lipid hydrophilic head boundary. Their structural impact on cytotoxicity is very weak. Hydrophilic surfactants such as C_12_E_12_ and C_12_E_23_ can only get inserted into the membrane in a small amount, with most surfactants disrupting the outer membrane layer to cause slow and weak cytotoxicity. These structural disruptions at the molecular level impose direct cytotoxic impacts as observed from the cells.

## 3 Conclusions

The irritancy of nonionic surfactants is a critical factor to consider for their applications in agri-spraying processes. Although the cytotoxicity of surfactants has been widely studied for decades,^22,25,31,33−38^ the interaction between surfactants and mammalian lipid membranes has not been systematically studied at the molecular level. Using a group of model nonionic surfactants, we have examined their interactions with both model and cell membranes. Biophysical studies using QCM-D, DPI, CLSM, AFM, and NR characterizations provided complementary structural features in the journey of nonionic surfactants embedded into cell membranes. The dynamic mode of irritations for varied nonionic surfactants was first revealed at the molecular level. Nonionic surfactants with well-balanced amphiphilicity can quickly fuse into the lipid bilayer, remove a large amount of lipids, and form transmembrane pores, leading to severe cytoplasm leakage and rapid cell killing. The membrane disruptive ability can be reduced by either increasing or decreasing the hydrophobicity of the surfactants significantly. Highly hydrophobic nonionic surfactants can weakly adsorb onto the lipid membrane with little interaction with the lipid membrane. Those with strong hydrophilicity can also be inserted into lipid membranes, but they mainly disrupt the outer lipid layer and cause weak and delayed cytoplasm leakage. These structural studies together with related physiochemical properties such as membrane leakage and permeability provide a useful basis for researchers to reduce the eye irritation potential of industrial formulations by controlling the cytotoxicity of nonionic surfactants and designing new surfactants as adjuvants in the future.

## 4 Materials and Methodology

### 4.1 Lipid and Nonionic Surfactants

DMPC (14:0 PC, 678 g/mol) and chain deuterated DMPC (dDMPC, 14:0 PC-d54) were purchased from Avanti Lipids (Alabaster, Alabama, USA) and used without further purification. Fluorescent lipid β-BODIPY FL C12-HPC [2-(4,4-difluoro-5,7-dimethyl-4-bora-3*a*,4*a*-diaza-*s*-indacene-3-dodecanoyl)-1-hexadecanoyl-*sn*-glycero-3-phosphocholine] was purchased from Thermo Fisher and used without further purification. Nonionic surfactants C_12_E_*m*_ (*m* = 2, 4, 6, 23) were purchased from Sigma-Aldrich, UK, and used without further purification. C_12_E_12_ was synthesized following the previously published method.^39^

All nonionic surfactants were freeze-dried under vacuum overnight before solutions were made up at concentrations of their associated CMCs. The CMC values for C_12_E_*m*_ (*m* = 2, 4, 6, 12, and 23) were used as previously reported as 0.04, 0.05, 0.07, 0.10, and 0.08 mM,^10,25^ respectively. For cell membrane permeability measurements, the surfactants were dissolved in phosphate-buffered saline (PBS, 10 mM PBS buffer, containing 137 mM NaCl, 2.7 mM KCl, 8.1 mM Na_2_HPO_4_, 1.9 mM KH_2_PO_4_) buffer. For QCM-D, DPI, and CLSM experiments, surfactants were dissolved in H_2_O. For NR characterizations, surfactants were dissolved in D_2_O and H_2_O.

### 4.2 Cell Culture and Cell Membrane Permeability Measurements

HCE cells (2.040 pRSV-T, a cell line, CRL-11516) were obtained from the American Type Culture Collection (ATCC; Manassas, VA, USA). The HCE cells were cultured at 37 °C in 5% CO_2_ in a keratinocyte-serum-free medium (GIBCO-BRL 17005-042). Supplements including 10 vol % heat-inactivated FBS, 5 ng/mL human recombinant epidermal growth factor (EGF), 0.05 mg/mL bovine pituitary extract, 0.005 mg/mL human insulin and 500 ng/mL hydrocortisone, 100U of streptomycin-penicillin, 5 mg/mL of gentamicin, and 0.25 μg/L amphotericin B were added to make the complete cell media. For cell proliferation, the HCE cells were grown on 75 cm^2^ flasks coated with 0.03 mg/mL rat tail collagen type I (First Link Ltd. 60-30-807).

To study the cell membrane permeability change induced by nonionic surfactants, three fluorescent dyes were used to stain the HCE cell nuclei, cytoplasm, and cytoskeleton separately as reported previously.^25^ The nuclei were stained blue with Hoechst 33342 (Thermo Fisher H1399). The cell plasma was stained in green by a CellMask Green plasma membrane indicator (Thermo Fisher C37608). The cytoskeleton was stained in red by Alexa Fluor 594 Phalloidin (Thermo Fisher A12381). Notably, Hoechst and plasma membrane indicators can penetrate the cell membrane while Phalloidin is not a cell membrane-permeable stain.

Briefly, 100 μL of HCE cell solution was preseeded in each well of a 96-well plate, at the concentration of 1 × 10^5^ cells/mL. After 24 h incubation, the cell media were replaced with 100 μL of different surfactant solutions (1 CMCs). Following incubation with the surfactants for 30 min, the surfactant solutions were replaced with 200 μL of the mixed stain solution containing Hoechst (2 μg/mL), Green plasma membrane indicator (1 μL/mL), and Phalloidin 594 (5 μL/mL, predissolved in methanol). After a 30 min incubation (avoiding light exposure), the stain solution was removed, and the cells were rinsed with PBS buffer. The cell nuclei, cytoplasm, and cytoskeleton were recorded through 350, 488, and 594 nm channels, respectively. Individual images taken from each channel from the same cell population were then merged by ImageJ software.

### 4.3 Preparation of Lipid Small Unilamellar Vesicles and the SLB

Two milligrams of DMPC lipids were first dissolved in chloroform, followed by solvent evaporation and freeze-drying overnight. The dry lipid film was then suspended in 1 mL of PBS buffer and sonicated for 15 min to form large multilamellar vesicles (LMVs). Small unilamellar vesicles (SUVs) were produced by extruding LMVs through a polycarbonate filter (prehydrated by PBS buffer, filter size: 50 nm) 31 times using a lipid extrusion apparatus.^40^ Notably, as the phase transition temperature for DMPC is 24 °C,^41^ the extrusion process was carried out on a hot plate under 40 °C (above the transition temperature).

The SUV solution was diluted to a concentration of 0.2 mg/mL in PBS buffer and then quickly deposited onto a clean silicon wafer to form the SLB at 40 °C (injection speed should be roughly 2 mL/min).^42^ The DMPC SLB was then rinsed with water at a speed of 2 mL/min to remove any undeposited SUVs. After rinsing, the temperature was lowered back to room temperature (20 °C). After a 20 min equilibrium, the system was ready for surfactant injection.

### 4.4 Quartz Crystal Microbalance with Dissipation

In this experiment, a Q-SENSE explorer with a flow module was utilized to dynamically trace the adsorption of different surfactants onto DMPC SLB. QCM-D measures the oscillation resonances of a crystal electrode when applied to a voltage. The frequency of the oscillation refers to the weight of the adsorbed layer, while the energy dissipation is viscoelastic energy losses due to changes in the sensing surface. The electrodes on the quartz sensor introduce a damping effect in which only the odd harmonics have the maximum movement at the surface, while the even harmonics coincide with a node. In practice, as harmonics go higher, the signal becomes weaker and the measurements become more susceptible to background noise. The first harmonic overtone is also significantly affected by the environment. Therefore, we only used few odd harmonics which give the most stable and strongest signals (third, fifth, and seventh).

The DMPC bilayer was formed via the fusion method and rinsed with plenty of buffer. After the overtones of the frequency shift (Δ*f*) and energy dissipation change (Δ*D*) reached equilibrium, the surfactants were injected into the sample chamber at a speed of 1 mL/min at a concentration of 1 CMC.

### 4.5 Dual Polarization Interferometry

An AnaLight Bio200 DPI system (Farfield Scientific, Inc., Crewe, UK) was used to obtain the optical properties of DMPC bilayers in the absence and presence of nonionic surfactants. An unmodified silicon oxynitride FB80 AnaChip (dimensions of 22 × 6 mm) made of four-layer dielectric stacks of silicon oxynitride on a silicon wafer surface was used. The chip contains two sample channels fabricated with a width of 1000 nm in the top silicon oxynitride layer. The chip was mounted on a chip holder and inserted into a temperature-controlled DPI sample chamber. The flow system connected to the DPI system comprised an autosampler (Farfield Scientific, Inc., Crewe, UK), a Harvard pump, and two 50 mL syringes with two three-way valves to control flow rates for the two sample channels individually. The laser (wavelength: 632.8 nm) propagates through the waveguides, producing the interference patterns. The spatial patterns of the interference fringes will be shifted when materials are deposited in the region of the evanescent wave field, which is on the surface of the top sample channels. The deposited materials change the refractive index within the evanescent field, altering the optical path length of the top waveguide and leading to interference pattern shifts. By splitting the incident light into two orthogonal polarizations (TE and TM waves), two individual measurements of the fringe shifts can be performed simultaneously.^43,44^

The SLB was generated as stated by using the fusion method. The nonionic surfactant solutions (1 CMC) were injected into the supported DMPC lipid bilayer at a speed of 1 mL/min for 3 min. After surfactant exposure, the SLB was rinsed with water at a speed of 1 mL/min until equilibrium. The refractive index and thickness of the adsorbed materials are coupled and the weight of the adsorbed layer is derived from the refractive index and thickness as shown below.^45^ The weight of the SLB, *W*, can be calculated from the thickness (*t*_s_) and refractive index (*n*_s_) combination as follows
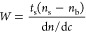
where *n*_b_ is the refractive index of the buffer and d*n*/d*c* is the gradient of a linear fit applied to the refractive index of a material solution as a function of its concentration (0.135 cm^3^/g for DMPC SLB).^26^ Thus, the refractive index of the adsorbed materials was fixed to be 1.47 (DMPC refractive index), and the weight and the birefringence of the adsorbed DMPC SLB were calculated spontaneously before, during, and after surfactant binding.^46^

### 4.6 Confocal Laser Scanning Microscopy

The interaction between surfactants and the fluorescent lipid membrane was traced by a Leica SP8 inverted confocal laser scanning microscope (Leica Microsystems GmbH, Wetzlar, Germany) equipped with a 63× oil immersion objective.^27^ Briefly, the C12-HPC SUV was produced using the extrusion method as described in Section 2.3. A droplet of C12-HPC SUV solution (10 μL) was quickly deposited onto a glass specimen and the lipid membrane was gently washed with PBS 2–3 times. Surfactants at a concentration of 1 CMC were then added. After 10 min, the sample was covered by a glass coverslip and characterized by confocal microscopy (excitation at 500 nm, emission at 513 nm). The images were analyzed by Imaris Cell Imaging software with appropriate contrast changes for better visualization.

### 4.7 Liquid Atomic Force Microscopy

AFM measurements of the model DMPC membrane in the presence of nonionic surfactants in an aqueous environment were performed on a Bruker Resolve (catalyst replacement). Briefly, 100 μL of DMPC SUV solution was quickly deposited onto a clean silicon wafer (1 cm × 1 cm). After 10 min of deposition, the solution was gently removed. 50 μL of nonionic surfactant solution was then added to the lipid membrane. The morphologies and phases of the lipid membrane against varied nonionic surfactants were acquired via the ScanAsyst Fluid mode using ScanAsyst Fluid plus probes.

### 4.8 Neutron Reflection

Neutron reflection studies were performed on the OFFSPEC beamline at the ISIS Spallation Neutron Faculty, Rutherford Appleton Laboratory using the previous setup.^16^ The *Q*-range was set from 0.008 to 0.4 Å^–1^ and the neutron beam illuminating region was approximately 3–4 cm^2^ defined by the slits setting. Each reflectivity profile consisted of measurements at two incident angles of 0.7 and 2.3°.

To achieve strong contrasts, tail-deuterated dDMPC and fully hydrogenated surfactants (denoted as hC_12_hE_4,6,12,23_) were used. Thus, two parallel contrasts were performed, including dDMPC with hC_12_hE_4,6,12, and 23_ in D_2_O and dDMPC with hC_12_hE_4,6,12, and 23_ in H_2_O. Characterization of the native silicon oxide surface was carried out at the very beginning of the experiments. The dDMPC was deposited onto the silicon oxide surface to generate the SLB as stated in Section 2.3. A quick measurement of bare dDMPC was performed in D_2_O and H_2_O to ensure the integrity of the SLB. The nonionic surfactant solutions were then injected into the DMPC SLB at a speed of 1 mg/mL. The molar volume and SLD for all materials used in this study are listed in Table 2. The reflectivity data were analyzed using Motofit software^47^ and a three-layer model for the DMPC SLB in the absence and presence of nonionic surfactants was established.

**Table 2:** SLD Values of All Materials Used in NR Experiments^a^

materials	D_2_O	H_2_O	hC_12_hE_*m*_	dDMPC tail	dDMPC head	Si	SiO_2_
molar volume Å^3^			350 + 63 m	808	297		
SLD ×10^–^^6^ Å^–^^2^	6.35	–0.56	0	6.9	0.75	2.07	3.47

a The SLD and molar volume for hC_12_hE_*m*_ are calculated from ref (12). The SLD and molar volume for the dDMPC tail and head are calculated from refs (48) and (49).

## Supporting Information Available

QCM-D and AFM results for C_12_E_4_ and C_12_E_23_ interacting with the model membrane; best-fit results from NR profiles for the DMPC lipid bilayer; and best-fit SLD results from NR profiles for C_12_E_4_, C_12_E_6_, C_12_E_12_, and C_12_E_23_ interacting with the model membrane (PDF)
