# Supplementary material for: Unraveling How Membrane Nanostructure Changes Impact the Eye Irritation of Nonionic Alkyl Ethoxylate Surfactants
Source: ACS Appl Mater Interfaces. 2023 Dec 11;15(50):59087–98. doi: 10.1021/acsami.3c14794 (PMC10739585; doi:10.1021/acsami.3c14794)
Supplement: Supplementary file 1 — am3c14794_si_001.pdf [file am3c14794_si_001.pdf]

Electronic supporting information

Unravelling How Membrane Nanostructure Changes Impact the Eye Irritation of Nonionic Alkyl Ethoxylate Surfactants

*Xuzhi Hu[a], Mingrui Liao[a], Kangcheng Shen[a], Ke Ding[a], Mario Campana[b], Sophie van der Kamp[c], Liz McInnes[c], Faheem Padia[c], Jian R. Lu[a]**

[a] Biological Physics Group, Department of Physics and Astronomy, School of Natural Sciences, University of Manchester, Oxford Road, Manchester, M13 9PL, UK.

[b] STFC ISIS Facility, Rutherford Appleton Laboratory, Didcot, OX11 0QX, UK.

[c] Syngenta, Jealott's Hill International Research Centre, Bracknell, Berkshire, RG42 6EY, UK.

* E-mail: j.lu@manchester.ac.uk. Tel: (+44)-161-200-3926

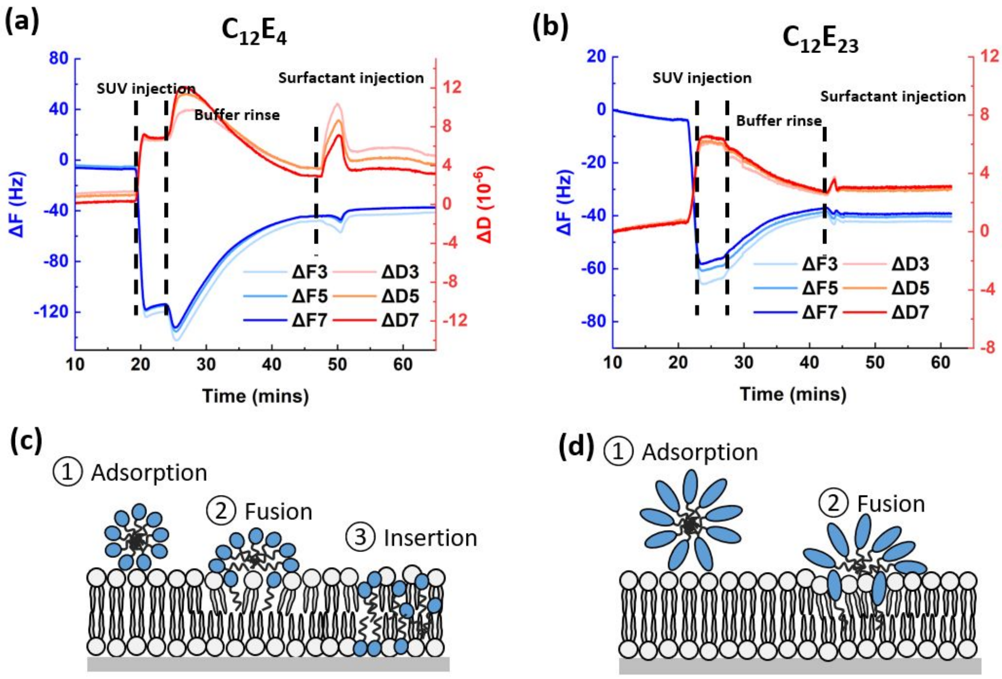

Figure S1: Resonance frequency overtones Δf and the energy dissipation overtones ΔD (n = 3, 5, 7) over time for (a) $C_{12}E_4$ and (b) $C_{12}E_{23}$ at a concentration of 1 CMC. Cartoon showing the kinetics of (c) $C_{12}E_4$ and (d) $C_{12}E_{23}$ binding with DMPC bilayers.

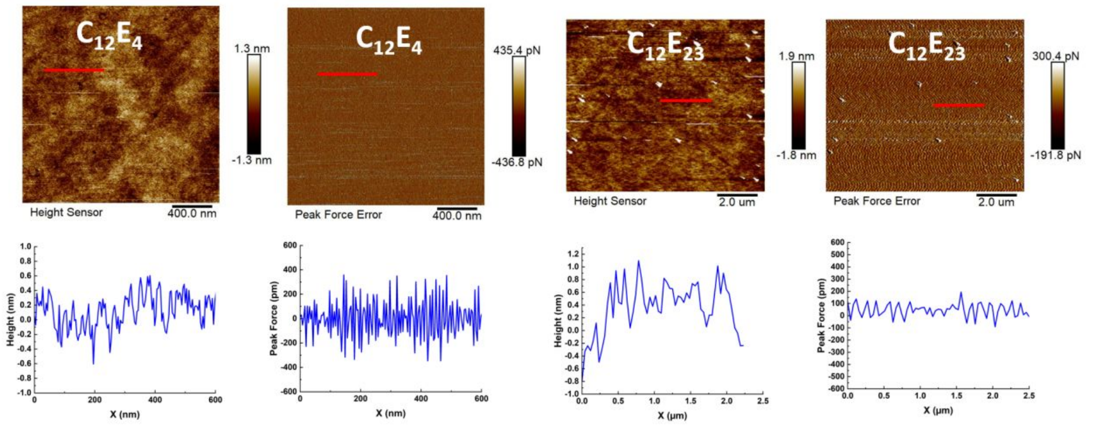

Figure S2: AFM images of lipid membranes in the presence of $C_{12}E_4$ and $C_{12}E_{23}$ surfactants in terms of height and peak force error.

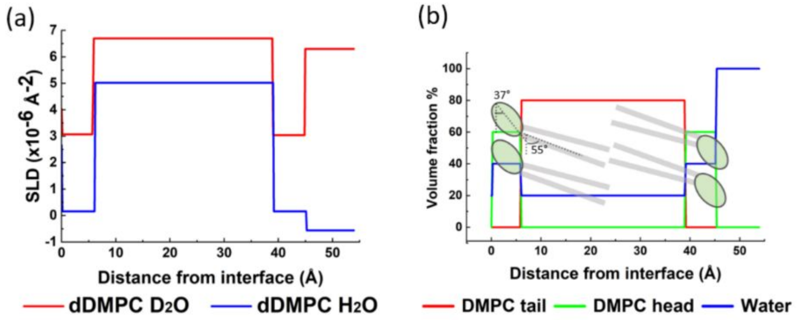

Figure S3: The best-fit results from NR reflectivity profiles for (a) SLD against distance from interface and (b) converted volume fraction against distance from the interface for DMPC tail, head and water.

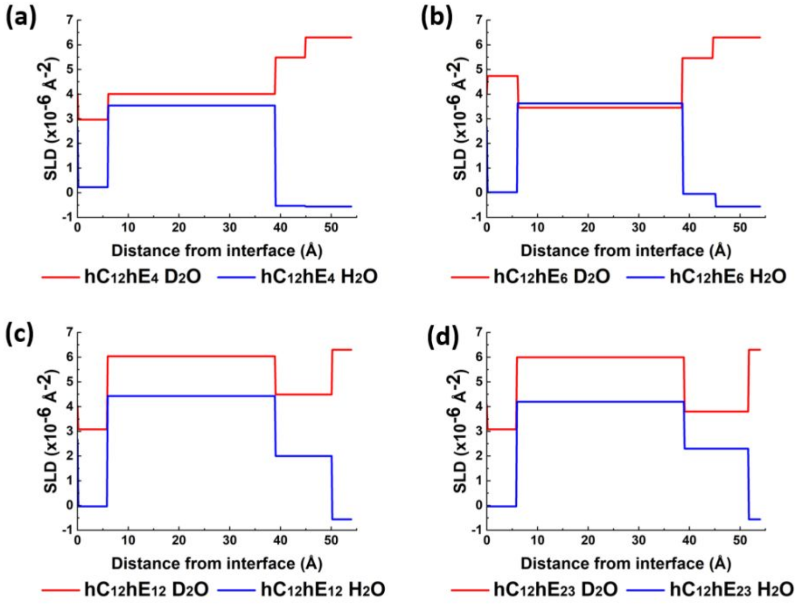

Figure S4: The best-fit results from NR reflectivity profiles for SLD against distance from interface for (a) $C_{12}E_4$, (b) $C_{12}E_6$, (c) $C_{12}E_{12}$ and (d) $C_{12}E_{23}$ surfactants.
